# Unfolding the empathic insights and tendencies among medical students of two gulf institutions using interpersonal reactivity index

**DOI:** 10.1186/s12909-024-05921-1

**Published:** 2024-09-09

**Authors:** Haniya Habib, Sara Anjum Niinuma, Khadeja Alrefaie, Heba Awad Al Khalaf, Mohammad Jasem Hani, Zeinab Yaareb Mosleh Al-Rawi, Zarish Hussain, Prianna Menezes, Sornali Rani Roy, Bincy Mathew, Salman Yousuf Guraya, Alfred Nicholson, Shaista Salman Guraya

**Affiliations:** 1 School of Medicine, Royal College of Surgeons in Ireland Bahrain, Busaiteen, 15503 Bahrain; 2 College of Medicine, University of Sharjah, Sharjah, 27272 United Arab Emirates; 3 University Hospitals of North Midlands NHS Trust, Stoke-on-Trent, ST4 6QG UK; 4 College of Medicine, Mohammed Bin Rashid University of Medical and Health Sciences, Dubai, 505055 United Arab Emirates

**Keywords:** Empathy, Medical students, Interpersonal reactivity index

## Abstract

**Background:**

Empathy is an essential core competency for future doctors. Unfortunately, the medical curriculum is infamously known to burn out aspiring doctors, which may potentially lead to a decline in empathy among medical students. This research was planned to understand the evolution of empathic approaches among students across the curriculum using the Interpersonal reactivity index (IRI) as a benchmark at the Royal College of Surgeons in Ireland - Medical University of Bahrain (RCSI-MUB) and University of Sharjah (UoS).

**Methods:**

We adopted a cross-sectional design and administered an online survey to the medical students of RCSI-MUB and UoS using a modified version of the IRI along with its three subscales of empathic concern (EC), perspective taking (PT), and personal distress (PD). To identify intra- and inter-institutional variations in empathy scores, the Analysis of Variance (ANOVA) was performed separately for each institution and with both institutions combined. A two-way ANOVA was conducted for the comparison between years and institutions. For the subscale analysis of EC, PT, and PD, we used one-way ANOVA for significant differences between years at both institutions. For the gender-effect analysis, t-test was performed to examine the differences in total IRI scores at both institutions combined and at each institution separately. Additionally, an Analysis of Covariance (ANCOVA) was done to identify the influence of gender on empathy scores.

**Results:**

A total of 140 students from both institutions participated in this study. We found a fluctuating pattern of empathy scores without a clear trend across the years. The sub-scales of EC, PD, and PT across academic years at both institutions showed significant differences within the EC at RCSI-MUB (*p* = 0.003). No significant differences were identified across other years from both institutions. There were significant differences between empathy scores from RCSI-MUB and UoS for EC (*p* = 0.011). Additionally, a pronounced interaction effect between year and institution was observed for PT (*p* = 0.032). The gender-wise analysis showed that female students had higher empathy scores than males (*p* = 0.004). The ANCOVA for IRI score results revealed a *p*-value of 0.023, indicating that gender plays a crucial role in empathy levels among medical students. The ANCOVA results revealed a *p*-value of 0.022 in the EC subscale.

**Conclusion:**

Our study unveiled intricate patterns in empathy development among medical students across years and genders at RCSI-MUB and UoS. These congruences and dissimilarities in empathy scores signal a subjective understanding of empathy by medical students. The disparities in understanding may encourage medical educators to embed empathy in standard medical curricula for better healthcare outcomes.

**Supplementary Information:**

The online version contains supplementary material available at 10.1186/s12909-024-05921-1.

## Background

In modern healthcare systems, empathy is considered as a fundamental pillar that plays a pivotal role in fostering patient trust, improving patient outcomes, and enhancing patient satisfaction [1]. Additionally, empathy allows physicians to communicate effectively with their patients and to express their humanistic and compassionate attitude [2]. Empathy pertains to the ability to perceive, recognize, and share another person’s feelings [3]. An empathic approach by physicians enhances physician-patient relationships, patient safety, and healthcare outcomes due to improved patient compliance and understanding of management plans [4]. Despite its outright benefits in the medical field, empathy needs to be better nurtured and understood in medical schools. A multitude of factors may contribute to this poor understanding of empathy, including a lack of a standard definition of empathy and consistency in the delivery and assessment of its cognitive, affective, and behavioral parts [5]. Furthermore, research has found that social commitment to medicine, including empathy, declines as students’ progress through their studies [6]. The medical curriculum is infamously known to burn out aspiring doctors, and consequently, their ethical values rapidly decrease, particularly during clinical years [7]. This is perhaps an aftermath of less emotional involvement of medical students with patients. [8].

In the context of patient care, a clear distinction between cognitive empathy, defined from a knowledge perspective (involving understanding processes), and empathy, defined from an emotional perspective (involving feelings and affect), is very crucial. These two forms of engagement yield different outcomes [9]. This emotional attunement of physicians fulfills the cognitive purpose of apprehending and sharing patients’ feelings and sufferings. Having a surplus of cognitive empathy (also known as clinical empathy) in patient care is consistently advantageous and can lead to the development of trust-based relationships, more precise diagnoses, enhanced patient compliance, and consequently, more favorable patient outcomes [10, 11]. However, an excess of emotional involvement, also known as sympathy, can be detrimental to patient care, resulting in emotional exhaustion and professional burnout among healthcare providers and unchecked emotional reliance on the part of the patient [12, 13]. Uncontrolled emotions can readily interfere with the objective process of making clinical decisions [14].

Empathy is contextually contingent and primarily shaped by situational factors and one’s inherent empathic tendencies [15]. These inherent tendencies can impact both cognitive and affective empathy. Individuals with high inherent empathic tendencies may better understand and appreciate others’ perspectives and emotions, complementing their cognitive empathy [16]. In medical education, understanding the inherent empathic tendencies of undergraduate medical students can provide valuable information to provide implications for their future patient care practices and interactions. In unison, such natural inclination towards emotional resonance can foster affective empathy, enabling one to genuinely share in the emotional experiences of others and respond compassionately [17]. Therefore, inherent empathic tendencies are integral to an individual’s overall empathic disposition, influencing how they connect with and understand the feelings and perspectives of those around them.

To date, numerous studies have explored the progression of empathy among medical students using various measurement scales, including the Interpersonal Reactivity Index (IRI) [18–22]. However, most of these studies have primarily focused on institutions in North America and Asia, with limited research conducted in the Middle Eastern region. It’s crucial to acknowledge that cultural nuances influence empathy, and therefore, findings from studies conducted in one cultural context may not necessarily generalize to medical institutions in other settings. This underscores the importance of conducting research in diverse cultural contexts to better understand the complexities of empathy development among medical students globally.

By investigating the empathic tendencies of medical students using the Interpersonal Reactivity Index (IRI) from two Middle Eastern institutions, we aim to shed light on the interplay of the complex relationship between innate empathy and external factors (educational environment), ultimately contributing to a more comprehensive understanding of empathy in medical education. By administering the IRI questionnaire, we aim to investigate the variations in empathic tendencies between these two groups of medical students, including perspective-taking (PT), empathic concern (EC), and personal distress (PD). PT measures the ability to shift to another person’s perspective, EC measures other-oriented feelings of sympathy and concern for others, and PD measures self-oriented feelings of personal anxiety and uneasiness in tense interpersonal settings. Additionally, this research seeks to identify potential factors or associations that may influence empathy scores within the context of medical education and institutional differences at the Royal College of Surgeons in Ireland, Medical University of Bahrain (RCSI-MUB) and the University of Sharjah (UoS) in the United Arab Emirates (UAE).

Our research delved into the evolution of the empathic approaches among medical students of two distinct academic institutions in the Middle Eastern region. The primary research question of our study was to determine the pattern of empathic insights of medical students across certain time points of their medical curriculum. A secondary end-point outcome was to compare yearly, gender-wise, and institutional variations in the understanding of medical students’ empathy between both institutions.

## Materials and methods

The Bachelor of Medicine and Bachelor of Surgery (MBBS) programs of RCSI-MUB and UoS contain a foundation year and a 5-year program with three phases of basic medical sciences, pre-clinical, and clinical sciences. Empathy is not delivered as a stand-alone subject in both institutions; however, it is arbitrarily covered during the clinical training of medical students. Between March and June 2023, an email invitation was sent to the undergraduate medical students of RCSI-MUB and UoS studying in foundation year till year 5. The invitation included details of the research study, a participant information leaflet (PIL), and a consent form. The registered students received another email with PIL and a SurveyMonkey questionnaire. Participants were requested to abide by the regulations for data privacy and their institutional codes of professional conduct throughout the study.

The study’s target population was undergraduate medical students who were currently studying foundation year till year 5 of study. A purposive sampling method was used to recruit medical students, and a convenience sample was obtained by approaching the participants who were available at the time of data collection. We invited student representatives from each year and institution to provide their perceptions of the IRI questionnaire. In total, we invited 144 student representatives from foundation to year 5 of both institutions, around 24 students from each year. Of those, 140 participated in our study with a response rate of 97%.

### Empathy measuring tools

An online survey was conducted using a modified version of the IRI, a widely recognized instrument for gauging empathy with a subset of scales and relevant tools [23]. This index was used for this study since it is the most widely used self-report measure for empathic tendencies due to its multidimensional approach and comprehensive assessment of empathic dispositions [24, 25]. Its validity and ease of administration are why we selected it for our study to assess empathic tendencies. The questionnaire also collected demographic data of student initials, gender, and year of study. We utilized the modified version of IRI, where we evaluated three of its four subscales: PT, EC, and PD, which contribute to cognitive and affective empathy [26] (Appendix I). PT, encompassing the cognitive aspect of empathy, delineates one’s capacity to understand and adopt another person’s viewpoint, thoughts, and feelings. On the other hand, EC is associated with affective empathy, encompassing the emotional resonance and compassionate response one feels in response to another person’s emotional distress or suffering. PD within the IRI pertains to an individual’s own discomfort and unease when confronted with the suffering of others, which can hinder empathic responses. Therefore, the IRI’s dimensions help dissect the intricate interplay between cognitive and affective empathy, shedding light on the multifaceted nature of empathic experiences. For the context of our study, we excluded the fantasy subscale, considering it less relevant to the medical milieu. The participants were instructed to answer on a 5-point scale of A-E ranging from ‘does not describe me well,’ ‘neutral’ to ‘does describe me well.’ Each subscale enquired about the participants’ insights on different empathic dispositions. A high score on PT indicates a tendency to adopt another’s psychological perspective, while a high score on EC shows a tendency to experience feelings of warmth, sympathy, and concern toward others. Finally, a high score on PD demonstrates a tendency towards feelings of discomfort when witnessing others’ negative experiences.

### Statistical analysis

#### Total IRI score analysis

Initially, we conducted an analysis of the total IRI scores across all participants from both institutions, stratified by academic year (foundation to year 5). This comprehensive approach provided an overarching insight into the empathic tendencies of students at different stages in their academic journey. Descriptive statistics, including mean and standard deviation, were calculated for each year group, offering a preliminary understanding of each cohort’s data distribution and central tendency. A One-way Analysis of Variance (ANOVA) was performed separately for each institution and with both institutions combined to discern whether significant differences in empathy levels existed between various years’ groups. This step was crucial for identifying intra- and inter-institution variations in empathy scores. Furthermore, a Two-Way ANOVA was conducted with ‘year’ and ‘institution’ as factors to elucidate any interaction effects between the academic year and the institution to determine whether institutions had differential impacts on students’ empathy levels across the years.

### Sub-scale analysis

Subsequently, we delved deeper into the individual sub-scales of the IRI (PT, PD, and EC) to dissect the components of empathy exhibited by students. Descriptive statistics for each sub-scale were computed for every year group at each institution, laying the groundwork for understanding the specific empathic tendencies prevalent in each cohort. One-Way ANOVA tests were employed for each sub-scale to probe for significant differences between years at both institutions. This granular analysis was important for unmasking the nuances of empathic development among students. Notably, since a significant variance was detected in the EC sub-scale at RCSI-MUB, post-hoc tests were executed exclusively for this group to identify any differences in insights about empathy. Additionally, Two-Way ANOVA tests were conducted for each sub-scale with ‘year’ and ‘institution’ as factors, facilitating a comparative analysis between the two institutions while considering the interaction effects.

### Gender effect analysis

To investigate the influence of gender on empathy, we calculated the mean and standard deviation of total IRI scores for each gender at both institutions. T-tests were performed to examine the differences in total IRI scores between genders at both institutions combined and the total scores of each sub-scale at each institution separately. The rationale for selecting the t-test was its suitability for comparing the means of two groups (male and female students). This step was essential for validating the gender effect on empathy levels, offering a lens through which the data could be interpreted from a gender perspective. Lastly, we included an Analysis of Covariance (ANCOVA). This was conducted to control the potential confounding effect of gender on empathy scores. ANCOVA was applied to the combined data from both institutions, integrating gender as a covariate. This step was crucial to discern if the observed variations in empathy scores, both in total IRI and its subscales (PT, PD, and EC), could be attributed to gender differences among student cohorts.

### Ethics approval

The study was approved by the relevant Institutional Research Ethical Committees of RCSI-MUB (REC/2023/147/18-Jan-2023) and UoS (REC-23-03-12-01-F). All participants gave fully informed written consent to participate at the start of the study.

## Results

### Influence of year and institution

A total of 140 medical students from RCSI-MUB and UoS responded to the online questionnaire in our study. There were 89 female and 51 male students. The yearly distribution of IRI and three sub-scales scores for all participating students from both institutions is presented in Table 1. This table illustrates the mean, median, standard deviation, and standard error of the total IRI scores and does not apply statistical tests to these values. Observationally, the data show a fluctuating but consistent pattern in empathy scores across the years without marked differences.

**Table 1:** Descriptive statistics for total interpersonal reactivity index (IRI) and subscales from RCSI-MUB and UoS (*N* = 140)

Total	Year	N	Mean	SE	Median	SD
IRI	Foundation	24	54.0	1.38	56.0	6.74
	Year 1	22	55.4	1.73	56.5	8.12
	Year 2	25	54.8	1.36	57.0	6.81
	Year 3	23	54.3	2.17	56.0	10.39
	Year 4	23	51.8	2.55	53.0	12.23
	Year 5	23	52.1	2.11	54.0	10.13
EC	Foundation	24	21.2	0.969	22.5	4.75
	Year 1	22	22.1	1.076	23.0	5.05
	Year 2	25	21.6	0.688	21.0	3.44
	Year 3	23	21.0	1.146	23.0	5.49
	Year 4	23	19.8	1.148	21.0	5.51
	Year 5	23	21.3	0.943	23.0	4.52
PD	Foundation	24	12.1	0.860	12.5	4.22
	Year 1	22	12.2	1.149	12.5	5.39
	Year 2	25	12.7	0.926	13	4.63
	Year 3	23	13.7	0.868	14	4.16
	Year 4	23	13.3	1.120	12	5.37
	Year 5	23	11.2	0.969	11	4.65
PT	Foundation	24	20.7	0.962	20.0	4.71
	Year 1	22	21.0	0.874	21.5	4.10
	Year 2	25	20.5	0.683	21	3.42
	Year 3	23	19.6	0.848	20	4.06
	Year 4	23	18.7	1.153	19	5.53
	Year 5	23	19.7	0.957	20	4.59

**Fig. 1:**
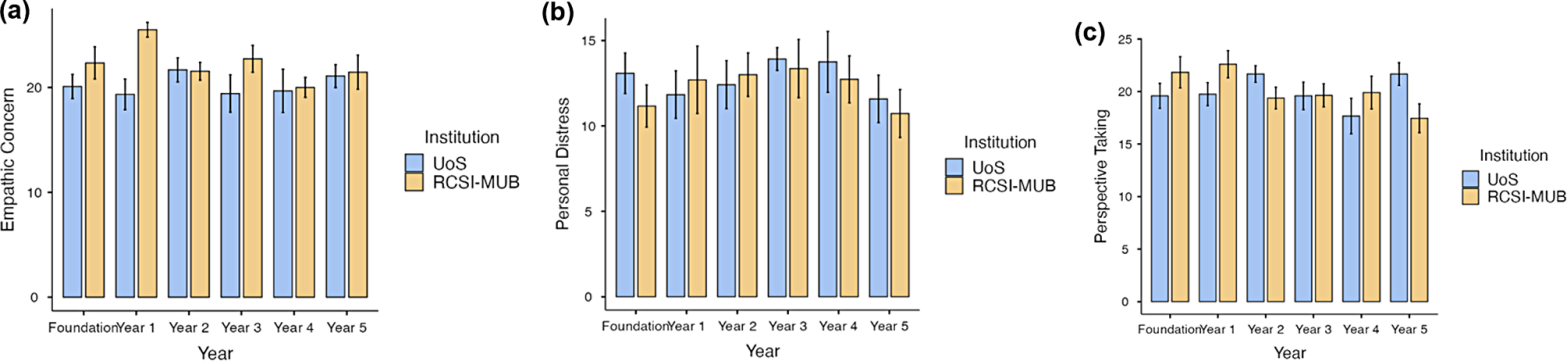
Bar plot based on descriptive data with mean scores for empathic concern (**a**), personal distress (**b**), and perspective taking (**c**)

Figure 1a and b, and 1c display the bar plots of mean scores for the EC, PD, and PT sub-scales, respectively, for students at RCSI-MUB and UoS across different academic years. The results of the One-Way ANOVA for all three IRI sub-scales across the academic years at both institutions showed significant differences within the EC sub-scale at RCSI-MUB (*p* = 0.003), as detailed in Table 2. Subsequently, the Tukey post-hoc test results, demonstrated in Table 3, show a significant pairwise difference in EC between Year 1 and Year 4 students at RCSI-MUB (*p* = 0.035). No significant differences were identified in comparison to other years from both institutions.

**Table 2:** One-way ANOVA for empathic concern, personal distress, and perspective taking from RCSI-MUB and UoS (*N* = 140)

	F	df1	df2	*p*
**RCSI - MUB**				
Empathic concern	4.824	5	28.5	**0.003**
Personal distress	0.525	5	28.3	0.756
Perspective taking	1.732	5	28.5	0.159
**UoS**				
Empathic concern	0.482	5	30.6	0.787
Personal distress	0.711	5	30	0.62
Perspective taking	1.418	5	30.5	0.246

**Table 3:** Results of the Tukey post-hoc test for empathic concern domain by RCSI-MUB students

		Foundation	Year 1	Year 2	Year 3	Year 4	Year 5
Foundation	Mean difference	—	-3.17	0.795	-0.394	2.33	0.8788
	*p*-value	—	0.468	0.997	1.000	0.746	0.995
Year 1	Mean difference		—	3.962	2.773	5.50	4.0455
	*p*-value		—	0.209	0.633	**0.035**	0.225
Year 2	Mean difference			—	-1.189	1.54	0.0839
	*p*-value			—	0.980	0.940	1.000
Year 3	Mean difference				—	2.73	1.2727
	*p*-value				—	0.625	0.977
Year 4	Mean difference					—	-1.4545
	*p*-value					—	0.960
Year 5	Mean difference						—
	*p*-value						—

**Fig. 2:**
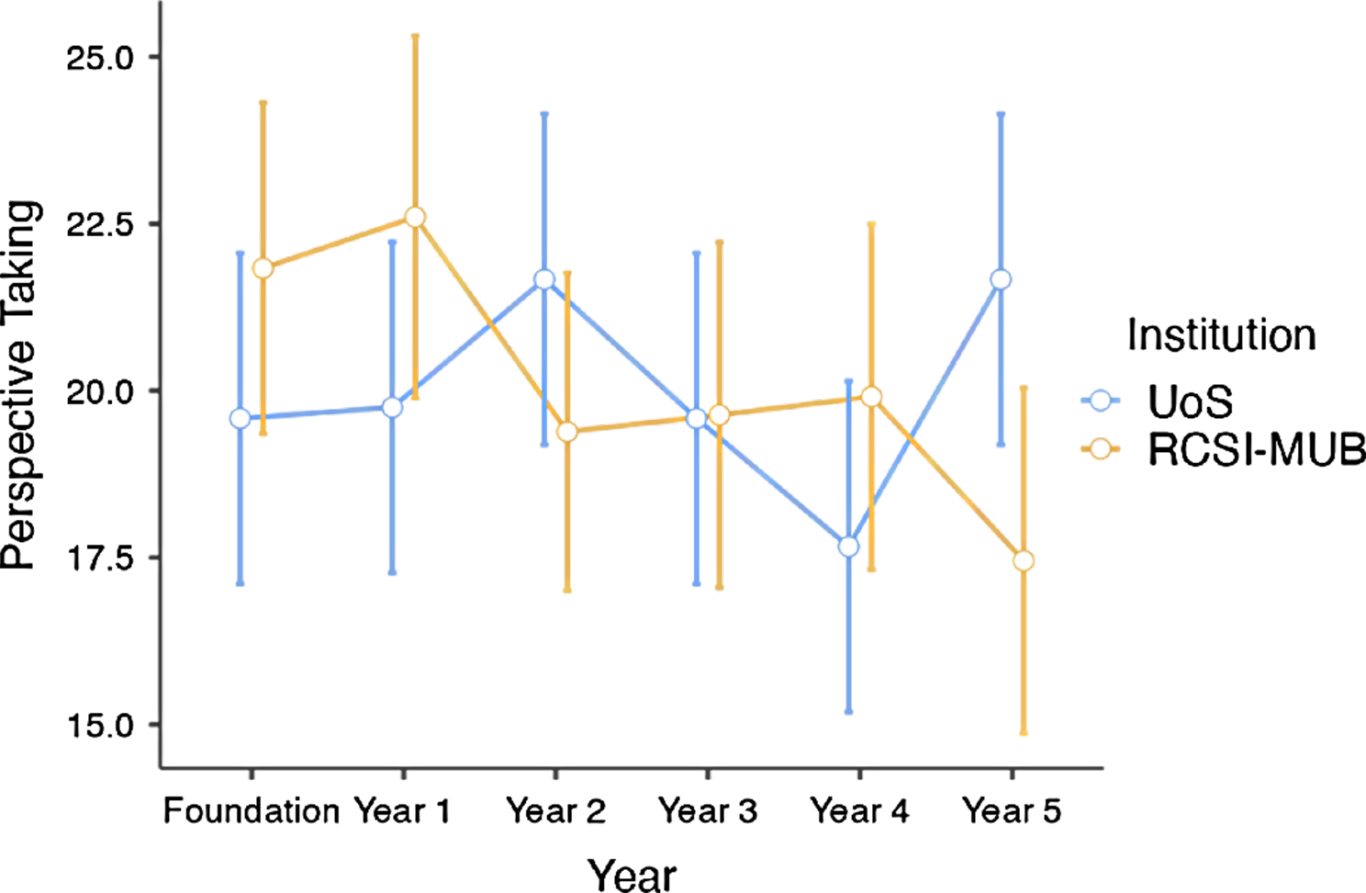
A plot diagram with the interaction effect between years and institution for perspective taking (*N* = 140)

Table 4 outlines the results of Two-Way ANOVA tests with significant differences between the insights of medical students from RCSI-MUB and UoS for EC (*p* = 0.011, Table 4). This implies that the educational environment or the mode of curricular delivery might exert a tangible influence on students’ empathic concerns. Additionally, a pronounced interaction effect between year and institution was observed for PT (*p* = 0.032, Table 4). An interesting analysis of the responses by medical students from RCSI-MUB and UoS for the subscale PT illustrates a unique pattern of the development of an empathic approach across different year groups (Fig. 2). Briefly, Fig. 2 displays the PT scores for foundation year students of RCSI-MUB students who exhibited higher scores than UoS. The PT scores of year 1 students at both institutions increased; however, a divergence was observed in year 2, with RCSI-MUB scores declining while UoS scores continued to increase. In year 3, the scores converged, with both institutions showing similar levels. Year 4 had a reversal, with RCSI-MUB scores increasing and UoS scores declining. Finally, in year 5, RCSI-MUB scores decreased while UoS scores escalated.

**Table 4:** Analysis of two-way ANOVA scores by years of study and institution (*N* = 140)

	Sum of squares	df	Mean square	F	*p*
**Empathetic concern**					
Year	79.6	5	15.9	0.724	0.607
Institution	146.5	1	146.5	6.661	**0.011**
Year^*^ institution	164.4	5	32.9	1.494	0.196
Residuals	2816.1	128	22.0		
**Personal distress**					
Year	89.44	5	17.89	0.767	0.575
Institution	8.13	1	8.13	0.349	0.556
Year^*^ Institution	31.86	5	6.37	0.273	0.927
Residuals	2984.26	128	23.31		
**Perspective taking**					
Year	90.150	5	18.030	0.9594	0.445
Institution	0.786	1	0.786	0.0418	0.838
Year^*^ Institution	237.460	5	47.492	2.5272	**0.032**
Residuals	2405.409	128	18.792		

### Gender-specific findings

Table 5 shows the percentages of male and female students across different years at RCSI-MUB and UoS and the overall gender distribution by institution. Table 6 compares the mean IRI scores for female and male medical students from RCSI-MUB and UoS using a t-test. The results showed that female students had higher overall empathy scores than males (*p* = 0.004). The gender-wise comparison of scores among medical students for EC, PD, and PT showed a significantly higher empathic concern by female students of RCSI-MUB than their male counterparts (*p* = 0.014), as shown in Table 7. This finding might have been influenced by the fact that all year 1 students at RCSI-MUB were females, potentially affecting the observed gender disparities. Table 8 outlines the ANCOVA for IRI results, which revealed a significant *p*-value of 0.023, which is below the conventional alpha level of 0.05, and ANCOVA for EC subscale shows a *p*-value of 0.022, affirming the impact of gender on empathy development.

**Table 5:** Percentages of male and female students across different academic years at RCSI-MUB and UoS

Institution	Academic year	Number of female students	Number of male students	Total students	% Female	% Male
RCSI-MUB	Foundation	9	3	12	75.00%	25.00%
	Year 1	10	0	10	100.00%	0.00%
	Year 2	9	4	13	69.23%	30.77%
	Year 3	9	2	11	81.82%	18.18%
	Year 4	4	7	11	36.36%	63.64%
	Year 5	7	4	11	63.64%	36.36%
	Total	48	20	68	70.59%	29.41%
UoS	Foundation	10	2	12	83.33%	16.67%
	Year 1	5	7	12	41.67%	58.33%
	Year 2	7	5	12	58.33%	41.67%
	Year 3	6	6	12	50.00%	50.00%
	Year 4	7	5	12	58.33%	41.67%
	Year 5	6	6	12	50.00%	50.00%
	Total	41	31	72	56.94%	43.06%

**Table 6:** Mean IRI scores for female and male medical students using t-test (*N* = 140)

	Gender	N	Mean	SE	Median	SD
IRI*	Female	89	55.4	0.985	57	9.29
	Male	51	50.8	1.156	52	8.25

*IRI: Interpersonal reactivity index

**Table 7:** Results of t-test for the scores between female and male medical students at RCSI-MUB

Domain	Statistics	df	*p*
Empathic concern	2.522	66	**0.014**
Personal distress	1.567	66	0.122
Perspective taking	-0.401	66	0.69

**Table 8:** Analysis of ANCOVA for IRI and subscales

IRI score both institutions combined					
	Sum of squares	**df**	Mean square	F	p
Year	194.5	5	38.9	0.482	0.790
Institution	46.5	1	46.5	0.576	0.449
Gender	427.5	1	427.5	5.293	**0.023**
Residuals	10258.8	127	80.8		
Perspective Taking					
Year	83.274	5	16.655	0.8798	0.497
Institution	0.489	1	0.489	0.0258	0.873
Gender	1.311	1	1.311	0.0693	0.793
Residuals	2404.098	127	18.930		
Personal Distress					
Year	98.9	5	19.79	0.865	0.507
Institution	17.5	1	17.54	0.767	0.383
Gender	78.6	1	78.64	3.437	0.066
Residuals	2905.6	127	22.88		
Empathic Concern					
Year	57.1	5	11.4	0.537	0.748
Institution	106.3	1	106.3	4.997	**0.027**
Gender	113.7	1	113.7	5.345	**0.022**
Residuals	2702.3	127	21.3		

## Discussion

The findings of our study offer a nuanced perspective on the trajectory of empathy development among medical students, reflecting a deeper understanding of empathy. Though there were insignificant differences for three subscales of IRI for each institution, there was a recognizable variation in EC scores and a fluctuating pattern of responses to PT between RCSI-MUB and UoS medical students. These results underscore the evolving nature of understanding empathy, that may be partly due to an absence of a standardized and accredited empathy-based curriculum. Lastly, female students had a significantly better understanding of EC, which signals a gender-based preference toward empathic care of patients.

These findings are consistent with the notion that empathy is not a static trait but rather a dynamic quality that evolves over time and can be influenced by various factors. In their cross-sectional and longitudinal mixed-methods study on undergraduate and graduate medical students, Michael et al., have deduced that targeted educational programmes should be introduced to develop empathic and patient-centered skills and competence of physicians [27]. Similar to other studies, our research also showed variations in responses and understanding of medical students in the absence of standard teaching of empathy in the curricula of both RCSI-MUB and UoS [28–30]. At the same time, we found yearly, gender, and institutional variations in understanding of empathy. The trends in PT scores suggested several points of consideration. The higher initial PT scores of foundation year students at RCSI-MUB compared to UoS may reflect differences in admission criteria, foundational training, or student characteristics between the two institutions. The shared increase in year 1 might indicate a common emphasis on developing perspective-taking skills early in medical education. The divergence in year 2, convergence in year 3, and subsequent variations may be indicative of differences in curricular focus, educational experiences, or other institutional factors that influence the development of perspective-taking skills at RCSI-MUB and UoS. The reversal in year 4 and the final intersection in year 5 may highlight variations in the later stages of medical training at each institution, potentially influenced by different clinical exposures or preparation for professional practice. These observed trends warrant further investigations to understand fully the factors contributing to the development of PT skills at medical academic institutions [31].

The identified significant variations within the EC sub-scale, particularly at RCSI-MUB between year 1 and 4 students, are particularly noteworthy. While the exact reasons for these variations require further exploration, these findings may indicate the uniqueness of the empathic development trajectory between years 1 and 4. Studies on empathy concerns among medical students report inconsistent data as they may decline, remain stable, or enhance [32, 33]. Piumatti et al. witnessed that empathy remains stable in most medical students and declines in fewer [34]. Furthermore, the authors observed that freedom to talk and patient-centric motives for studying medicine were associated with a higher and consistent empathic approach. The differences in EC scores among students of both institutions might indicate variations in educational environment or curriculum or both. Further research is essential to interpret the implications of these findings fully and understand the factors contributing to the observed differences in EC scores among medical students at RCSI-MUB and UoS.

The significant interaction effect between year and institution for PT suggests that the journey of empathy development is not linear and is influenced by a myriad of factors, including the educational environment. The gender differences observed, especially within RCSI-MUB, further complicate the narrative. The exclusive female composition of year 1 students at RCSI-MUB could have introduced a potential bias, potentially skewing the results. However, gender distribution was more consistent in some years, particularly at UoS. The ANCOVA results revealed a *p*-value of 0.023, which falls below the conventional alpha level of 0.05, and *p*-value of 0.022 for EC subscale. This finding indicates that gender influences empathy levels among medical students. Female students exhibited higher empathy scores than their male counterparts, suggesting that gender differences might be an important factor to be considered in medical education and training. This insight into the gender disparities in empathic tendencies can be pivotal for medical educators and curriculum designers, as it highlights the need for tailored approaches to develop and nurture empathy among future healthcare professionals. However, we acknowledge the limitations in our demographic analysis due to the unavailability of additional sociodemographic details such as age, nationality, and socioeconomic status.

Most published studies have reported that female medical students are more empathic than their male counterparts [32, 35–43]. However, despite the overwhelming evidence supporting this correlation, there have been inconsistencies in the findings of some studies. Electroencephalography measures have not found significant gender differences in empathic abilities [44]. A cross-sectional study in Pakistan yielded results that align with the general trend, showing that females had significantly higher scores on specific items of the IRI and EC scales [45]. Nevertheless, when considering total empathy scores, both male and female students demonstrated similar levels of empathy overall. This emphasizes the importance of diverse participation in research to ensure comprehensive insights. Developing PT skills and strategies to mitigate PD are fundamental core competencies of medical graduates. Empirical research has argued that medical students’ distress may potentially lead to cynicism and subsequently affect their care of patients and their relationship with peers and faculty [46]. The manifestations and causes of PD, alongside its potential adverse personal and professional outcomes, are detrimental to enhancing EC among medical students [47]. These adverse consequences can be arrested by targeting medical education and paying more attention to fortifying the EC of medical students.

In our exploration of empathic development among medical students, the year-based analysis did not show significant differences across academic years, indicating consistency in empathy levels as students’ progress through their medical education. This finding adds an intriguing dimension to our understanding of empathy, suggesting that despite varying challenges and experiences encountered in different stages of medical education, the overall capacity for empathy among students remains relatively stable. However, this finding may be due to the cross-sectional nature of the study.

Despite significant similarities in the core curricula at RCSI-MUB and UoS, we posit that other factors unique to each institution, such as cultural contexts, teaching methodologies, student demographics, and extracurricular activities, might influence the development of empathic behaviors. Our study, therefore, recommends that researchers extend beyond the curriculum to include these broader institutional factors, offering a more nuanced perspective on how empathy is shaped within medical education settings.

Despite the significant role of empathy in enhancing healthcare outcomes, this important trait in medical students and residents has paradoxically been reported to decline during their clinical training [13, 48]. Several factors can contribute, such as emotional exhaustion, suboptimal social support, burnout due to workload, and an inadequate curriculum [49]. For the professional enhancement of empathic skills of medical students, educators can consider well-structured faculty development programs [50], interprofessional education [51], simulation-based scenarios [52], and patient-centered medical education for effective communication [27]. Particular attention must be paid to interprofessional education, which carries great potential to enhance the empathic concerns of medical students [53].

Medical institutions might contemplate implementing structured empathy training modules, ensuring that future doctors are equipped with this indispensable soft skill. The observed differences between institutions underscore the need for a tailored approach, considering the unique characteristics of each institution. As the medical community continues to recognize the importance of empathy in patient care, research like ours calls for the need for continuous evaluation and refinement of medical curricula to foster this critical trait.

### Strengths and limitations

This study was conducted on medical students of two premier medical institutions of the Middle Eastern region. This unique opportunity allowed us to analyze the cross-cultural and curricular influence of empathic approaches of medical students across the entire continuum of medical education. Additionally, this research yields significant findings that medical educators can use to modify the medical curriculum.

Our study has several limitations. First, based on the nature of the study, the number of participants may be considered small, limiting the generalizability of the findings. Second, this study identified differences in empathic approaches at defined time points rather than in a prospective manner. Due to the cross-sectional design, the research measured different participants at distinct stages rather than following the same individuals over time. Consequently, the findings reflect differences in empathy scores between separate groups rather than changes within the same individuals. This design limitation means that the study captures variations in empathy approaches at specific time points rather than longitudinally tracking how individual empathy develops or changes throughout progression in a medical program. Third, the results may not be used to cover other cultures or contexts. Finally, the self-reported insights of students to IRI may reflect subject bias. Individuals are likely to overestimate their empathy due to factors like social desirability.

### Future directions

Our study used a self-administered IRI questionnaire and did not explore the empathy that takes place between patients and medical students. Future investigators should employ studies that could focus on patient perceptions of empathic student and physician behavior. Furthermore, expanding the sample size and incorporating longitudinal examination of participants to observe changes over time will certainly advance the understanding of medical students’ empathy. In addition, future research could benefit from incorporating gender balance and sociodemographic variables to present a more comprehensive demographic profile and to understand their potential influences on empathy development among medical students.

## Conclusion

In summary, our study substantially contributes to the evolving nature of empathy development among medical students and the potential impact of curriculum and gender on this critical attribute. Though there are certain variations in insights about empathy, this study observed a unique fluctuating trend between RCSI-MUB and UoS across years and gender. Such disparities highlight the potential ramifications of curricular elements, teaching methodologies, clinical experiences, or even institutional ethos on students’ empathy development. This research urges medical educators to modify existing medical curricula by inculcating empathy into standard teaching and learning pedagogies.

## Electronic supplementary material

Below is the link to the electronic supplementary material.

Supplementary Material 1

## Data Availability

The datasets used and/or analyzed during the current study are available from the corresponding author upon reasonable request.

## Abbreviations

IRI: Interpersonal Reactivity Index
RCSI-MUB: Royal College of Surgeons in Ireland – Medical University of Bahrain
UoS: University of Sharjah
MBBS: Bachelor of Medicine and Bachelor of Surgery
EC: Empathic concern
PT: Perspective taking
PD: Personal distress
ANOVA: Analysis of Variance
ANCOVA: Analysis of Covariance
PIL: Participant Information Leaflet
