# Supplementary material for: Unfolding the empathic insights and tendencies among medical students of two gulf institutions using interpersonal reactivity index
Source: BMC Med Educ. 2024 Sep 9;24:976. doi: 10.1186/s12909-024-05921-1 (PMC11385142; doi:10.1186/s12909-024-05921-1)
Supplement: Supplementary file 1 — Supplementary Material 1 [file 12909_2024_5921_MOESM1_ESM.docx]

# **Questionnaire Before Discussion**

The following statements inquire about your thoughts and feelings in a variety of situations. For each item, indicate how well it describes you by choosing the appropriate letter on the scale at the top of the page: A, B, C, D, or E. When you have decided on your answer, fill in the letter next to the item number. READ EACH ITEM CAREFULLY BEFORE RESPONDING. Answer as honestly as you can.

ANSWER SCALE

A B C D E

*Does Neutral Does Not Describe Describe*

*Me Well Me Well*

1. I often have tender, concerned feelings for people less fortunate than me.

2. I sometimes find it difficult to see things from the "other guy's" point of view.

3. Sometimes I don't feel very sorry for other people when they are having problems.

4. In emergency situations, I feel apprehensive and ill-at-ease.

5. I try to look at everybody's side of a disagreement before I make a decision.

6. When I see someone being taken advantage of, I feel kind of protective towards them.

7. I sometimes feel helpless when I am in the middle of a very emotional situation.

8. I sometimes try to understand my friends better by imagining how things look from their perspective.

9. When I see someone get hurt, I tend to remain calm.

10. Other people's misfortunes do not usually disturb me a great deal.

11. If I'm sure I'm right about something, I don't waste much time listening to other people's arguments.

12. Being in a tense emotional situation scares me.

13. When I see someone being treated unfairly, I sometimes don't feel very much pity for them.

14. I am usually pretty effective in dealing with emergencies.

15. I am often quite touched by things that I see happen.

16. I believe that there are two sides to every question and try to look at them both.

17. I would describe myself as a pretty soft-hearted person.

18. I tend to lose control during emergencies.

19. When I'm upset at someone, I usually try to "put myself in his shoes" for a while.

20. When I see someone who badly needs help in an emergency, I go to pieces.

21. Before criticizing somebody, I try to imagine how I would feel if I were in their place.
